# The Anæsthetist's Outfit

**Published:** 1910-12-31

**Authors:** 


### Anæsthetics

### THE ANÆSTHETIST'S OUTFIT.

The following list is that of articles which should invariably be at hand or be taken to every case: Stethoscope; anæsthetics and apparatus for administration; mouth props; mouth wedge; gags; sponges; sponge holders; tongue forceps; needle and silkworm gut; tracheotomy instruments; hypodermic syringe and drugs. The following may be added with advantage, especially if at all likely to be wanted: cylinder of oxygen with tubing, etc.; instruments and tabloids for saline infusion.

*Stethoscope.*—It is perfectly true that more may often be learnt as regards the patient's fitness for an anæsthetic by observing the breathing, colour, and pulse than by listening at the chest. Valvular disease producing loud bruits may have no obvious result, provided there is good compensation and the anæsthetisation skilfully conducted. On the other hand, dangerous forms of cardiac degeneration may not be revealed by the most careful auscultation. Still, the stethoscope should always be used, even when the above reservations are borne in mind, and even when it seems obviously unnecessary. Its use may reveal something unsuspected, and gives confidence to the patient and friends, whilst if it be omitted and ill effects occur from any reason whatever the anæsthetist may be severely blamed.

*Apparatus.*—Care should be taken to see this is in good working order and thoroughly clean, and that there is a sufficient quantity of anæsthetic. The anæsthetist should always be prepared with one or more alternatives, which will depend upon the age of the patient, the nature of the operation, and other circumstances.

*Mouth Props.*—For extraction of teeth these are usually supplied and inserted by the operator, but it is well to use one (if a gag be not inserted before beginning) in the case of mouth-breathers, and when the teeth are so complete that difficulty may arise in opening the mouth, either for operation or to relieve obstructed breathing. For such purposes a small wooden prop in the shape of a bobbin or double wedge should be inserted horizontally, so that the bicuspids close on the constricted part—then if the mouth be slightly opened or the lower jaw has to be pushed forward it will not fall out.

*Mouth Wedge.*—This, in its simplest and perhaps its best form, is merely a stout boxwood handle tapering to a wedge, by which the molars can be separated for easier insertion of a gag. It should not be used (as the writer has seen done) for levering the lower jaw forwards to improve the breathing—the jaw should be pushed forwards by a thumb or finger at its angles.

*Gags.*—Some form of Mason's gag is required, one with short handles being more generally useful. For removal of tonsils, etc., two gags, for use on alternate sides, will facilitate quick operating if a Doyen's be not preferred. A screw gag for forcible and rapid opening may be needed in emergencies.

Coarse *sponges* entangle clots and mucus better than fine ones. Care must be taken that there are no tags which may come off.

*Sponge Holders* with sharp teeth and rings are untrustworthy. The former are apt to tear away from the sponges and the latter to become displaced by the edges of the teeth. Long-handled serrated forceps are preferable.

*Tongue Forceps.*—The usual pattern with broad rings is recommended. When clamped there should be space between them to hold the tongue without *crushing* it. These, however, should only be used for drawing out the tongue or holding it out momentarily—not for keeping it forward for any length of time, which is best done by a piece of gut passed through it a quarter of an inch from the tip—this can be looped over the anæsthetist's finger or held by an assistant. Most of the forceps which pierce the tongue bruise it as well. Much distress may result from damage to the tongue, causing continued pain and interfering with talking and taking nourishment.

*Tracheotomy instruments* may never be required during a lifetime, but, on the other hand, they may be badly wanted.

The *Hypodermic syringe* must be overhauled occasionally if not in constant use, or it probably will be found out of order when required.

Drugs, such as atropin, morphine, pituitary extract, will of course vary somewhat, according to the practice of the anæsthetist.

*Oxygen and materials for saline infusion* should always be provided in any case where there is possibility of shock, whether from initial exhaustion, prolongation of the operation, hæmorrhage, or any other reason. The former is needed when respiratory failure may occur, as in operations on the brain.

